# Curative Treatment of POMP-Related Autoinflammation and Immune Dysregulation (PRAID) by Hematopoietic Stem Cell Transplantation

**DOI:** 10.1007/s10875-021-01067-7

**Published:** 2021-06-16

**Authors:** Andrea Meinhardt, Paula C. Ramos, R. Jürgen Dohmen, Nadja Lucas, Min Ae Lee-Kirsch, Benjamin Becker, Jan de Laffolie, Tomás Cunha, Tim Niehues, Ulrich Salzer, Ayami Yoshimi, Miriam Erlacher, Anke M. J. Peters, Stephan Ehl, Brigitte Strahm, Carsten Speckmann

**Affiliations:** 1 Center for Pediatrics and Adolescent Medicine, Medical Center, University Hospital Giessen, Giessen, Germany; 2 Institute for Genetics, Center of Molecular Biosciences, Department of Biology, Faculty of Mathematics and Natural Sciences, University of Cologne, Cologne, Germany; 3 Department of Pediatrics, Medical Faculty and University Hospital Carl Gustav Carus, Technische Universität Dresden, Dresden, Germany; 4 Center for Dermatology and Allergology, Medical Center, University Hospital Marburg, Marburg, Germany; 5 Center for Pediatrics and Adolescent Medicine, Helios Hospital Krefeld, Krefeld, Germany; 6 Institute for Immunodeficiency, Center for Chronic Immunodeficiency (CCI), Faculty of Medicine, Medical Center-University of Freiburg, Freiburg, Germany; 7 Center for Pediatrics and Adolescent Medicine, Department of Pediatric Hematology and Oncology, Faculty of Medicine, Medical Center-University of Freiburg, Freiburg, Germany

To the Editor:

We describe our clinical and laboratory observations in a patient with “Proteasome-Related-Autoinflammatory Disease” (PRAID), caused by a de novo variant in *Proteasome maturation protein* (*POMP*). Hematopoietic stem cell transplantation was curative and reversed the clinical and hematopoietic phenotype of our patient.

Proteasome maturation protein (POMP) functions as a chaperone, that is essential for assembly of the 20S proteasome and the immunoproteasome [1]. The proteasome is a central non-lysosomal proteolytic structure important for degrading polyubiquitinated proteins. Upregulation of POMP by interferons (IFN) enhances major histocompatibility complex (MHC)-I dependent antigen presentation and thus the specific T-cell response [2]. Frameshift variants in *POMP* escaping nonsense-mediated decay (NMD) are associated with “Proteasome-Related-Autoinflammatory Diseases” (PRAID), whereas autosomal recessive deletions in the 5´-UTR cause “Keratosis Linearis with Ichthyosis Congenita and sclerosing Keratoderma” (KLICK) syndrome, an intrinsic dermatological disorder [3, 4]. PRAID are characterized by early-onset immunodeficiency and autoimmunity in combination with non-infectious skin lesions; a presentation also termed CANDLE (chronic atypical neutrophilic dermatosis with lipodystrophy and elevated temperature) syndrome [3]. Four PRAID [3, 5, 6] and 14 KLICK syndrome [4] patients were previously reported (summarized in Online Resource 1).

Current therapeutic strategies in PRAID include corticosteroids, other immunomodulators, and biologics, e.g., interleukin (IL)-1ß-blocking agents or tumor necrosis factor (TNF)-α-inhibitors [3, 7]. While Janus-kinase (JAK) 1/2 inhibition was reported to be efficacious in various interferonopathies [7], its therapeutic potential in PRAID remains unknown. Whether hematopoietic stem cell transplantation (HSCT) can be a curative treatment option is another open matter of debate since POMP is ubiquitously expressed also in non-hematological tissues. Hitherto, HSCT outcome data was reported in only one patient [5]. Yet, this patient carried an additional pathogenic mutation in *minichromosome maintenance complex component 3 associated protein* (*MCM3AP*), resulting in a variant pre-HSCT hematological and cellular phenotype with myelodysplasia, genomic instability, and normal B-cell development in the bone marrow [5]. Two further PRAID patients were mentioned to be referred for HSCT, without further reported details [3].

We describe our observations in a male Caucasian patient (P1), in whom HSCT resulted in sustained remission of all PRAID associated symptoms (Fig. 1A). P1 presented at the age of 2 months with erosive erythematous skin lesions and thrombocytopenia. Shortly after, he acquired *Pneumocystis jirovecii* pneumonia (PJP), from which he recovered, but which prompted further evaluations for a suspected inborn error of immunity (IEI). Immunophenotyping identified elevated CD3+ T-cells (6.627/µl), which were predominantly CD4+ (5.549/µl). Total NK (42/µl) and B-cells (131/µl) were reduced, with a relative increase of plasmablasts and immunoglobulin (Ig)A + switched memory B-cells. Serum analyses showed dysgammaglobulinemia with elevated IgA (9.5 g/l) and IgM (3.0 g/l). T-cell proliferation was reduced after mitogen stimulation with PHA and not responding after stimulation via the T-cell receptor with anti-CD3 (± CD28) (data not shown).

**Fig. 1:**
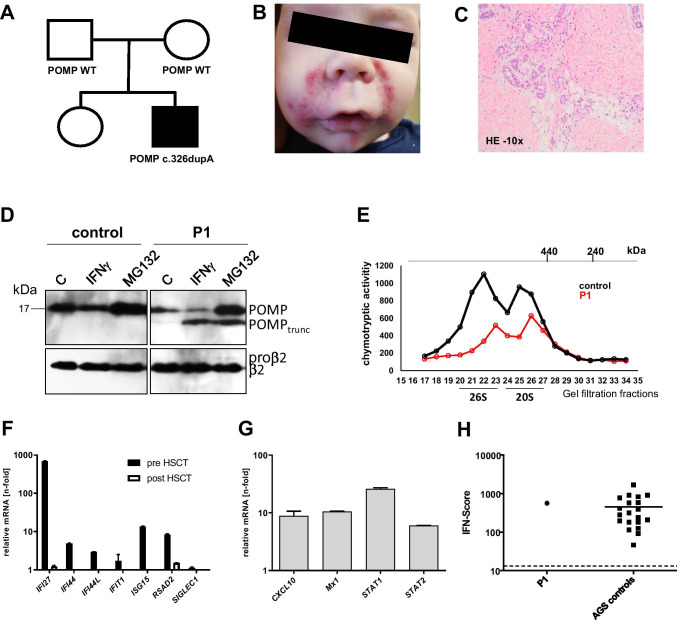
Clinical and experimental overview of P1. (**A**) Pedigree of P1. (**B)** Clinical skin phenotype before HSCT. (**C**) Hematoxylin and eosin staining of a skin biopsy (10x magnified) showing perivascular lympho-histiocytic infiltrates. (**D**) Immunodetection of POMP and its truncated version (POMP_trunc_), precursor (proβ2), and mature forms of subunit β2 (PSMB7). Control (C) and patient (P1) fibroblasts were treated with 150 units/mL IFNγ (Peprotech 300–02) and 1 µM MG132 (Sigma-Aldrich C2211) for 24 h. Proteins were extracted in 50 mM Tris pH 7.5 buffer containing 2 mM ATP, 5 mM MgCl_2_, 1 mM DTT, and 15% glycerol. From each sample, 10 μg of protein were analyzed by SDS-PAGE(12%) and western blotting (PVDF) with 1:1000 anti-POMP monoclonal antibody (D2X9S, Cell Signaling Technology) as well as anti-β2 monoclonal antibody (MCP168 ENZO Life Sciences). Beta 2 protein was immunostained as loading control. (**E**) Comparison of the chymotryptic activity profiles of proteins extracted from control and patient fibroblasts proteins after gel filtration fractionation (superose 6; flow rate 0.3 mL/min; fraction volume 0.5 mL). The column was calibrated with ferritin (440 kDa) and catalase (240 kDa). (**F**) RT-PCR analysis of interferon-stimulated genes (before and 1.8 years post-HSCT) in PBMC and (**G**) fibroblasts. RNA extraction as well as expression analysis of the indicated genes, using quantitative real-time RT-PCR, was carried out as previously reported [10]. (**H**) Strongly increased IFN score in PBMC of P1 vs. Aicardi-Goutières syndrome (AGS controls). The score was calculated as previously described [10]. In short, gene expression of IFN-stimulated genes was measured by quantitative real-time polymerase chain reaction in the presence of a calibrator and normalized to the expression of two housekeeping genes. For comparison with healthy individuals, an IFN score of 12.49 (dashed line) indicates the median IFN score of 10 healthy controls plus 2 SD

Between 4 and 12 months, recurrent non-infectious erosive papulovesiculous skin lesions (Fig. 1B) and conjunctivitis were chief complaints, which improved under systemic corticosteroid treatment. Histology revealed predominant perivascular lympho-histiocytic infiltrates (Fig. 1C), an already previously reported feature of PRAID [3]. Starting at 10 months, P1 failed to thrive. Endoscopy revealed ubiquitous lympho-plasmacellular mucosal inflammation and superinfection with astro- and norovirus aggravated the clinical course from 14 months onward.

Trio exome sequencing identified an unreported heterozygous de novo *POMP* variant in P1 (c.326dupA; p.Asp109Glufs*2) in exon 5, predicted to lead to expression of a C-terminally truncated version of POMP lacking 32 of its 141 residues. Biochemical studies in fibroblasts confirmed expression of a truncated POMP polypeptide of predicted size along with a full-length POMP protein; the truncated POMP was absent in lysates from control fibroblasts (Fig. 1D). Upon treatment of fibroblasts with IFN-γ, levels of the truncated POMP form were increased, which was in contrast to the full-length form of POMP, which was reduced in extracts from both control and P1. This is in line with an earlier report showing that POMP is encased by the nascent 20S proteasome and degraded upon completion of the assembly and activation process [8]. Upon IFN-γ stimulation, POMP is degraded faster, apparently because assembly of the immunoproteasome is faster than that of the housekeeping counterpart [9]. The higher metabolic stability of the truncated form of POMP indicates that it does not participate in the formation of active proteasome particles. In contrast to IFN-γ stimulation, treatment with the proteasome inhibitor MG132 led to elevated levels of both full-length and truncated POMP (Fig. 1D). The conclusion that truncated POMP does not promote the formation of active proteasomes is in line with the observation that proteasome activity was reduced in P1 compared to control cells (Fig. 1E).

The inflammatory phenotype of PRAID has been shown to be related to a pathological upregulation of type I interferons. The exact molecular mechanism by which dysfunctional POMP triggers type I IFN responses remains to be elucidated. It has been assumed that a disturbed proteasome function results in accumulation of ubiquitinated proteins, leading to endoplasmic reticulum stress and secondary type I IFN upregulation [3]. Quantitative RT-PCR analysis from peripheral blood mononuclear cells (PBMC) and fibroblasts of P1 (Fig. 1F and G) confirmed a markedly increased expression of IFN-stimulated genes (ISG), consistent with a constitutive type I IFN activation. The calculated IFN score was even comparable to scores of patients with Aicardi-Goutières syndrome (AGS controls, Fig. 1H), a prototypic type I interferonopathy caused by a defect in the DNA/RNA sensing pathway.

P1 developed aggravated, transfusion-dependent microcytic anemia and thrombocytopenia from 12 months onward. His peripheral blood showed a left shift of the myeloid lineage with immature myeloid cells and eosinophilia. Bone marrow showed hyperplastic, yet not reduced, megakaryocytes with moderate dysplasia and disturbed maturation of the myeloid lineage (displayed in Online Resource 2). Flow cytometry of bone marrow detected a maturation arrest on the level of pre-BI- to pre-BII-progenitor cells (displayed in Online Resource 2), explaining the peripheral B-cell lymphopenia.

In light of progressive bone marrow dysfunction and chronic susceptibility to viral and opportunistic infections, P1 was evaluated for HSCT, which was carried out at 19 months following reduced toxicity conditioning (thiotepa, treosulfan, fludarabine, and serotherapy with anti-thymocyte globulin) from a 10/10 HLA-matched unrelated donor. The course was uneventful with timely engraftment and full donor chimerism from day 30 onward. Skin lesions resolved with the beginning of chemotherapy and previous chronic norovirus infection cleared. Thirty-one months after HSCT, the patient is off any medication and demonstrates normal psychomotor development, normalization of weight and height, and remission from his previous inflammatory and immunodeficiency phenotype. All immunological parameters are normalized (summarized in Online Resource 3), including T-cell proliferation and IFN signature analysis from PBMC (Fig. 1F). Western blot analysis from PBMC obtained 31 months after HSCT further showed that the previously truncated POMP was successfully replaced by a full-length POMP form (displayed in Online Resource 4), compatible with full donor chimerism.

Similar to four previously reported patients, our patient showed a severe and potentially life-threatening manifestation of PRAID in infancy [3, 5, 6]. Immunosuppressants, the current mainstay of treatment, may transiently improve immune dysregulatory symptoms but bear the risk of long-term toxic side effects and may even further increase the patients’ inborn susceptibility to infections. Our data demonstrate that HSCT has the potential to fully correct the inflammatory and immunodeficiency phenotype of PRAID. Following HSCT, we observed no critical non-hematological manifestations, suggesting that our patient’s phenotype was primarily driven by POMP-deficient cells of hematological origin.

## Supplementary information

Online Resource 1 (DOCX 32 kb)

Online Resource 2 (PDF 450 kb)

Online Resource 3 (DOCX 24 kb)

Online Resource 4 (PDF 32 kb)
