# Supplementary material for: Curative Treatment of POMP-Related Autoinflammation and Immune Dysregulation (PRAID) by Hematopoietic Stem Cell Transplantation
Source: J Clin Immunol. 2021 Jun 16;41(7):1664–7. doi: 10.1007/s10875-021-01067-7 (PMC8452576; doi:10.1007/s10875-021-01067-7)
Supplement: Supplementary file 1 — (DOCX 32 kb) [file 10875_2021_1067_MOESM1_ESM.docx]

**Online Resource 1. Clinical description and laboratory findings in our (P1) and previously reported POMP mutated patients (P2-19)**

|  | P1 | P2 | P3 | P4 | P5 | P6-19 |
| --- | --- | --- | --- | --- | --- | --- |
| Clinical description |  |  |  |  |  |  |
| Sex | male | female | male | male | male | male, female |
| Age of onset | 2 mo | 3 mo | 2nd wk | 2nd wk | birth | n.a. |
| Infections |  |  |  |  |  |  |
| Opportunistic | PJP | PJP | PJP | - | - |  |
| Diarrhea | prolonged, noro-, astrovirus |  | pers., norovirus | prolonged, astrovirus | - |  |
| Respiratory tract | rec, viral, URT |  | rec, viral, LRT^1^,  pulmonary nodules | viral, LRT³,myco- bact. lung infection | - |  |
| Other | - | meningitis, syst. atyp. myco-bacteriosis | bacteremias^2^ | bacteremia^4^ | rec. FUO,  chronic lymphadenitis |  |
| Dystrophy | yes | yes | n.a. | n.a. | yes |  |
| Other symptoms | lid edema, unilateral |  |  | seizures | painful joints,  sc nodules, violaceous eye lids, periorbital edema, development delay  dysmorphic features^5^ |  |
| Skin abnormalities | rec. papulo-vesicular | rec. papulous | persistent erythe-matous lesions with central ulceration | persistent erythe-matous lesions with central ulceration | annular plaques | palmoplantar keratoderma, linear hyperkeratolytic plaques, ichthyosi-form scaling |
| Histology | perivascular lympho-histiocytic infiltrates | leukocytoclastic vasculitis | neutrophilic dermatosis  microthrombosis | neutrophilic dermatosis | leukocytoclastic vasculitis | unspecific lympho-histiocytic infiltrates |
| Treatment |  |  |  |  |  |  |
| HSCT | yes (RIC) | yes (RIC) | no details reported | no details reported | n.a. | no |
| Age at HSCT | 1.6 y | 3.4 y | n.a. | n.a. |  |  |
| Follow up | 2.5 y, healthy | 4 y, healthy | n.a. | n.a. | 10 y, alive |  |
| Laboratory investigations | |  |  |  |  |  |
| thrombocytopenia | progressive, | intermittent | intermittent  mild | cyclic  moderate | - |  |
| other hematological   abnormalities | progressive anemia | anemia (pers., mild)  rec. monocytopenia | n.a. | n.a. | - |  |
| bm changes | Progressive ineffective hematopoiesis,  B cell maturation arrest | myelodysplasia, normal B cell development | n.a. | n.a. | n.a. |  |
| B cells  plasmabl., mem.B | low  elevated | absent to low  n.a. | very low  elevated | very low  elevated | n.a. |  |
| T cells (CD3+)  CD4+  CD8+ | elevated  elevated low | normal  elevated (>2y) | elevated  elevated low | elevated  elevated low | n.a.  n.a.  n.a. |  |
| T cell proliferation | reduced | reduced | n.a. | n.a. | n.a. |  |
| Nk cells | low | absent to low | normal | normal | n.a. |  |
| Ig | IgA, M elevated | IgG, A, M low (3 mo), later IgA elevated | IgG, A, E elevated | IgG, A, E elevated | n.a. |  |
| Autoantibodies | - | n.a. | ANA+ anti-B2-GP I+ anti-thyroid ab +  DAT | ANA +  anti-B2-GP I+ anti-thyroid ab + | ANA+ |  |
| POMP mutation | c.326dupA | c.333_334delTA | c.334_335delAT | c.342_348delinsACC | c.344_345insTTTGA | c.-95delC (5´UTR) |
| Reference | this report | Gatz et al; Human mutation 2016 | Poli et al., Am J Hum Gen 2018 | Poli et al., Am J Hum Gen 2018 | Brehm et al., J Clin Invest 2015; Megarbane et al., J Rheum 2002 | Takeichi and Akiyama, Front Immunol 2020 |

ab = antibodies; ANA = anti-nuclear antibodies; anti-B2-GP I = anti-b2 glykoprotein I; atyp. =atypical; bm = bone marrow; DAT = direct anti-globulin test; FUO = fever of unknown origin; HSCT = allogenic hematopoietic stem cell transplantation; Ig = immunoglobulins; LRT = lower respiratory tract; mem.B = memory B cells; mo = month; n.a. = data not available; pers. = persistent; PJP = Pneumocystis jirovecii Pneumonia; rec. = recurrent; plasmabl. = plasmablasts, RIC = reduced intensity conditioning; sc = subcutaneous; transf.dep. = transfusion dependent; syst = systemic; URT = upper respiratory tract; wk = week of life; y = years; ^1^ Adenovirus, RSV (respiratory syncytial virus), Parainfluenza; ² MRSA (methicillin-resistent Staphylococcus aureus, Klebsiella pneumoniae, Pseudomonas, gram negative rods; ^3^ rhinovirus; ^4^ salmonella; ^5^ large nasal bridge, large interphalangeal articulations, brachydactyly
