# Supplementary material for: Curative Treatment of POMP-Related Autoinflammation and Immune Dysregulation (PRAID) by Hematopoietic Stem Cell Transplantation
Source: J Clin Immunol. 2021 Jun 16;41(7):1664–7. doi: 10.1007/s10875-021-01067-7 (PMC8452576; doi:10.1007/s10875-021-01067-7)
Supplement: Supplementary file 2 — (PDF 450 kb) [file 10875_2021_1067_MOESM2_ESM.pdf]

Online Resource 2

I)

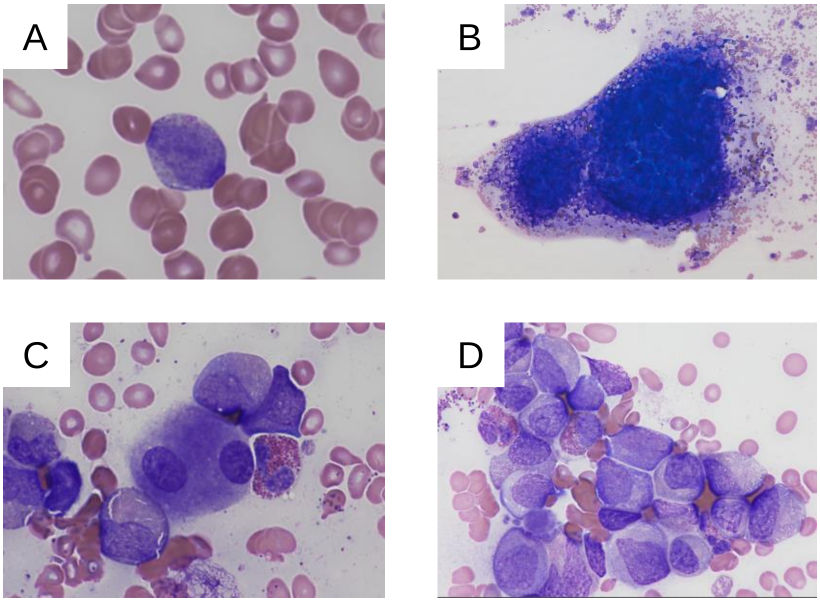

II)

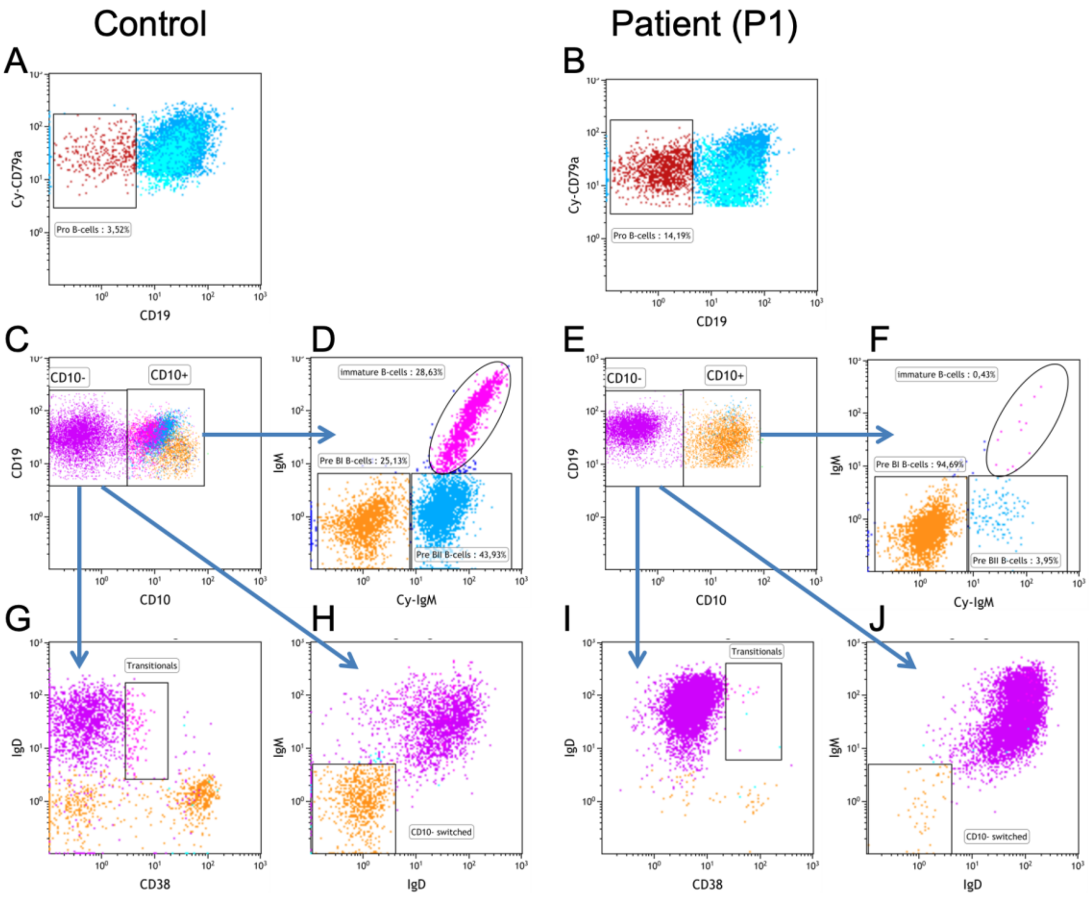

I) Blood and bone marrow morphology A) Left shift of peripheral blood with presence of myeloid precursors B) hyperplastic bone marrow C) a dysplastic megakaryoblast with round seprated nuclei D) strongly left-shifted myelopoiesis and increased eosinophils

II) Analysis of mature B cells and B-cell precursors in bone marrow by flow cytometry. A,B. Analysis of Pro B-cells (CD19-CD79a+) gated on CD3/16/33-CD79a+ cells. **C,E.** Analysis of mature B cells (CD19+CD10-) and B-cell precursors (CD19+CD10+) gated on CD3/16/33-CD19+ cells. **D,F.** Analysis of B-cell precursor subpopulations: Pre-BI (IgM-cyIgM-), PreB-II (IgM-cyIgM+)and immature B-cells (IgM+cyIgM+) gated on CD19+CD10+. **G,H,I,J.** Analysis of mature B-cell subpopulations gated on CD19+CD10-. cyCD79a, cytoplasmatic CD79a. cyIgM, cytoplasmatic IgM.
