# Supplementary material for: Curative Treatment of POMP-Related Autoinflammation and Immune Dysregulation (PRAID) by Hematopoietic Stem Cell Transplantation
Source: J Clin Immunol. 2021 Jun 16;41(7):1664–7. doi: 10.1007/s10875-021-01067-7 (PMC8452576; doi:10.1007/s10875-021-01067-7)
Supplement: Supplementary file 3 — (DOCX 24 kb) [file 10875_2021_1067_MOESM3_ESM.docx]

**Online Resource 3: Comparison of blood parameters pre- and post-HSCT in P1**

| **parameter**  (normal range for 2-5 years) | **pre-HST** | | **post-HST**  (+2.6 years) |
| --- | --- | --- | --- |
|  | 2-3 mo. of age | 12 mo of age |  |
| **Blood count**   - Leucocytes (5400-13800/µl)   - Granulocytes (1500-8500/µl)   - Lymphocytes (2200-8500/µl) - Hb (10.7 -13.9 g/l) - Thrombocytes (200.-460.000/µl) | 8700/µl  3040/µl  4330/µl  9.1 g/dl  52.000/µl | 3700/µl  930/µl  2170/µl  7.3 g/dl  31.000/µl | 6700/µl  3050/µl  3180/µl  11.4 g/dl  266.000/µl |
| **CRP** (<1 mg/l) | <0,5 mg/l | 44,13 mg/l | <0.5 mg/l |
| **Immunoglobulines**   - IgG (3.5-11.8 g/l) - IgM (0.3-1.0 g/l) - IgA (0.4-1.9 g/l) | 9.2 g/l  1.2 g/l  6.0 g/l | 12.9 g/l  1.4 g/l  9.5 g/l | 8.3 g/l  1.0 g/l  1.0 g/l |

| **Lymphocyte subsets**   - Lymphocytes total - CD3+ (1400-3700/µl) - CD4+ (700-2200/µl) - CD8+ (490-1300/µl) - CD4/CD8 Ratio (1.1-1.8) - CD19+ (390-1400/µl) - CD16+56+ (130-720/µl) - HLADR+ in CD4+ (40-180/µl; 2-6%) - HLADR+ in CD8+ (55-420/µl, 2-20%) - CD45RA+ in CD4+ (430-1500/µl, 53-86%) - CD45R0+ in CD4+ (220-660/µl; 9-26%) - IgD+CD27-naïve B-cells (88-100%) - IgD+CD27+ of B-cells (2-43%) - IgD-CD27+ of B-cells (0.9-29%) - IgA+CD27+memory B-cells - IgM+/-CD38++ plasmablasts of B-cells - IgD-IgM+CD27+ of B-cells (IgM only; 0.1-23%) | 6856/µl  6627/µl  5549/µl  614/µl  9,03  131/µl  42/µl  3,2%  0,7%  92.7%  6%  48.01%  11.48%  15.05%  8.19%  20.63%  2.9% | 3640/µl  2460/µl  1710/µl  601/µl  2.84  887/µl  290/µl  31/µl  80/µl  1343/µl  338/µl  63%  6.8%  14.13%  6.99%  5.73%  4.07% | 3180/µl  2299/µl  1593/µl  532/µl  2.99  576/µl  269/µl  42/µl  57/µl  1176/µl  353/µl  73.97%  9.43%  6.79%  1.53%  0.12%  3.59% |
| --- | --- | --- | --- |
| **T-cell proliferation**   - after PHA - after T-cell dependent stimulation  (anti-CD3 +/- CD28) |  | decreased  absent | normal  normal |
| **Vaccination titers**   - against tetanus - against diphtheria - against hepatitis B virus - against measles, mumps, rubella, chickenpox |  | protective  non-protective  non-protective  - | protective  protective  protective  protective |
