# Supplementary material for: Curative Treatment of POMP-Related Autoinflammation and Immune Dysregulation (PRAID) by Hematopoietic Stem Cell Transplantation
Source: J Clin Immunol. 2021 Jun 16;41(7):1664–7. doi: 10.1007/s10875-021-01067-7 (PMC8452576; doi:10.1007/s10875-021-01067-7)
Supplement: Supplementary file 4 — (PDF 32 kb) [file 10875_2021_1067_MOESM4_ESM.pdf]

Online Resource 4

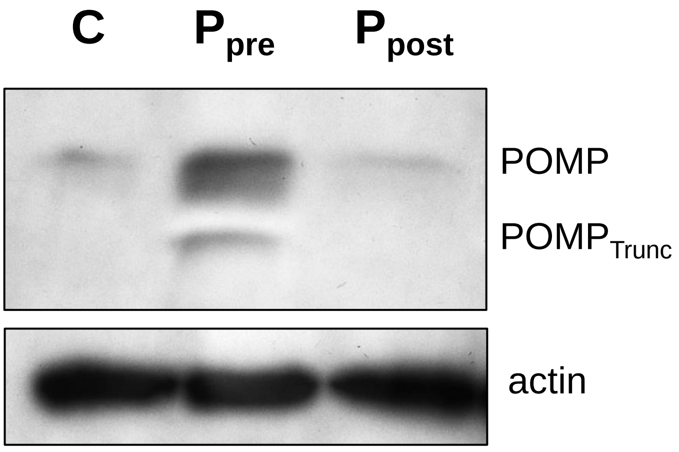

Immunodetection of POMP in PBMC. An analysis prior to HSCT (P_{pre}) confirms the presence of truncated POMP ($POMP_{trunc}$), as detected in P1's fibroblasts (Figure 1). Analysis of PBMC taken 31 months post HSCT (P_{post}) demonstrates full and sustained correction with POMP expression comparable to a healthy and age-matched control (C). The higher level of POMP/truncated POMP in the patient sample prior to HSCT is most likely reflecting an Nrf-mediated feedback response leading to increased expression of genes encoding proteasome subunits and POMP as a consequence of the reduced efficiency of proteasome assembly and maturation (Ebstein et al., Frontiers Immunol. 201; doi: 10.3389/fimmu.2019.02756), or reflecting type-I-IFN induction of POMP expression (Heink et al., PNAS 2005; DOI: 10.1073/pnas.0501711102), or both.
